# Different Risk of Tuberculosis and Efficacy of Isoniazid Prophylaxis in Rheumatoid Arthritis Patients with Biologic Therapy: A Nationwide Retrospective Cohort Study in Taiwan

**DOI:** 10.1371/journal.pone.0153217

**Published:** 2016-04-11

**Authors:** Tsai-Ling Liao, Ching-Heng Lin, Yi-Ming Chen, Chia-Li Chang, Hsin-Hua Chen, Der-Yuan Chen

**Affiliations:** 1 Department of Medical Education and Research, Taichung Veterans General Hospital, Taichung, Taiwan; 2 Institute of Biomedical Science and Rong Hsing Research Center for Translational Medicine, National Chung Hsing University, Taichung, Taiwan; 3 Department of Health Care Management, National Taipei University of Nursing and Health Sciences, Taipei, Taiwan; 4 Division of Allergy, Immunology and Rheumatology, Taichung Veterans General Hospital and Faculty of Medicine, National Yang Ming University, Taipei, Taiwan; 5 Institute of Microbiology and Immunology, Chung Shan Medical University, Taichung, Taiwan; The Catholic University of the Sacred Heart, Rome, ITALY

## Abstract

Increasing evidence indicates an increased risk of tuberculosis (TB) for rheumatoid arthritis (RA) patients receiving biologic therapy, and the effectiveness of isoniazid prophylaxis (INHP) in TB prevention. We aimed to examine 1) the incidence rate (IR) and risk factors for TB among RA patients receiving different therapies; 2) INHP effectiveness for TB prevention; 3) mortality rates after TB diagnosis in patients receiving different therapies. This retrospective study was conducted using a nationwide database: 168,720 non-RA subjects and a total of 42,180 RA patients including 36,162 csDMARDs-exposed, 3,577 etanercept-exposed, 1,678 adalimumab-exposed and 763 rituximab-exposed patients. TB risk was 2.7-fold higher in RA cohort compared with non-RA group, with an adjusted hazard ratio (aHR) of 2.58. Advanced age, male, the use of corticosteroids≧5mg/day, and the presence of diabetes mellitus (DM), chronic obstructive pulmonary disease and chronic kidney disease were risk factors for developing TB. Using csDMARDs-exposed group as reference, aHR of TB was the highest with adalimumab treatment (1.52), followed by etanercept (1.16), and the lowest with rituximab (0.08). INHP could effectively reduce TB risk in biologics-exposed patients. Mortality rates after TB diagnosis were higher in RA patients, particularly the elderly and those with DM, with lower rates in adalimumab-exposed patients compared with csDMARDs-exposed patients. In conclusion, TB risk was increased in patients receiving TNF-α inhibitors, but the risk associated with rituximab therapy was relatively low. With the effectiveness of INHP shown in the prevention of biologics-associated TB, stricter implementation of INHP should be beneficial. The mortality from biologics–associated TB may be efficiently reduced through increased awareness.

## Introduction

Tuberculosis (TB) remains a major global public health issue nowadays, as an estimated 9.0 million people developed TB and 1.5 million died from the disease in 2013 [1]. In Taiwan, the mandatory Bacillus Calmette-Guérin (BCG) vaccination was implemented extensively for newborn babies as well as 7~10-year-old school children without a characteristic BCG scar, and the vaccination coverage had reached 97.0% [2]. Our previous hospital-based study also showed approximately 97.9% of RA patients had received BCG vaccination [3]. However, Taiwan sustains a high TB prevalence, despite the extensive implementation of well-known TB control measures [4]. For rheumatoid arthritis (RA) patients, the risk of developing TB is particularly high, possibly due to disease-related immune dysregulation or the immunosuppressive effects of therapeutic agents [5–7].

Rheumatoid arthritis-related comorbidities such as diabetes mellitus (DM), and chronic kidney disease (CKD) may also affect TB risks [8–10]. Increasing evidence indicates that the risk of active TB is further elevated for patients receiving corticosteroids or tumor necrosis factor (TNF)-α inhibitors therapies [9–14]. The guidelines have recommended that effective TB screening should be carried out and isoniazid prophylaxis (INHP) be initiated before anti-TNF-α therapy if latent TB infection (LTBI) is detected [15].

Rituximab, an anti-CD20 monoclonal antibody, has been shown to be effective for RA patients with inadequate response to anti-TNF-α therapy [16]. Although previous studies demonstrated that B cells serve a role in the host defense against *M*. *tuberculosis* infection [17], active TB has not been reported from RA patients receiving rituximab therapy in clinical trials [18] or in real-world practice [19], with only 3 cases of active TB reported in a survey conducted by the Emerging Infections Network (EIN) [20].

The prevalence of TB is higher in Asian population than in the United States (US) or Europe [1, 5]. However, few Asian population-based epidemiological studies have investigated the effect of INHP on biologics–associated TB prevention among RA patients receiving different therapeutic agents. In view of that, we utilized a nationwide database, NHI Research Database (NHIRD) for this research. The National Health Insurance (NHI) program in Taiwan is a mandatory universal health insurance program that provides comprehensive medical care to more than 99% of the population [7,21], and its database, NHIRD, is confidentiality maintained according to the guidelines of the Bureau of NHI [22]. Herein, we examined the incidence rate and risk factors for TB, as well as the death rates after TB diagnosis and their risk factors among RA patients receiving different therapies, including conventional synthetic disease-modifying antirheumatic drugs (csDMARDs), TNF-α inhibitors, and rituximab.

## Materials and Methods

### Data Source and study design

**Fig 1:**
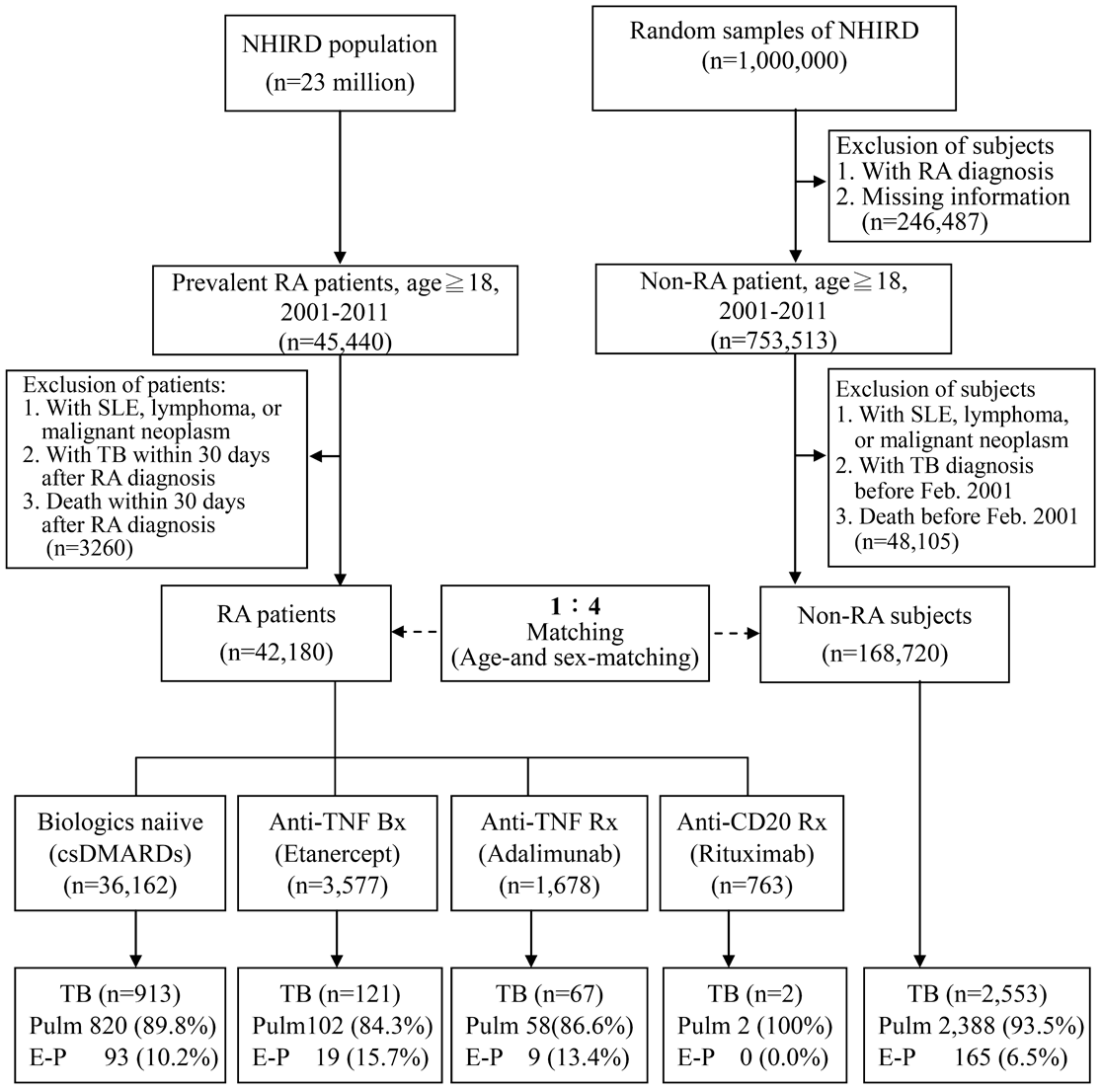
Flow chart of case selection in this study. The rheumatoid arthritis (RA) patients, and age- and sex-matched non-RA control subjects were selected from the Taiwan National Health Insurance research database (NHIRD).

This retrospective population-based cohort study was conducted using 2001–2011 claims data retrieved from NHIRD, which consists of detailed health care information from more than 23 million enrollees, representing more than 99% of Taiwan’s entire population. The Longitudinal Health Insurance Database (LHID) 2000 contains all the original claim data of 1,000,000 individuals randomly sampled from Registry for Beneficiaries of the NHIRD released by the NHRI, which confirmed that the random samples were representative of the general population in Taiwan (Fig 1). Personal information including weight, height, family history, laboratory examination results, lifestyle and habits such as smoking and alcohol use, is not provided by the NHIRD. This study was approved by the Institutional Review Board of Taichung Veterans General Hospital (CE13151-1).

### Patients

Patients with RA were identified primarily by using the International Classification of Diseases, Ninth Revision, Clinical Modification (ICD-9-CM) codes. In addition, with a registry system for catastrophic illnesses in the NHIRD, which includes RA certified by two rheumatologists, we ensured that the diagnosis of RA (ICD-9-CM code 714.0) was made according to the 1987 American College of Rheumatology criteria [23] and the Registry of Catastrophic Illness Database (RCIPD) of the NHIRD.

The definitions of comorbidities were made according to the published literature and based on ICD-9-CM codes [7–8, 21], including DM (ICD-9-CM code 250), liver cirrhosis (ICD-9-CM code 571), chronic obstructive pulmonary disease (COPD, ICD-9-CM codes490-492 and 496), and CKD (ICD-9-CM codes 585). All comorbidities were defined by their presence of disease in three or more medical visits prior to the index date. The Charlson comorbidity index (CCI) was calculated according to the adaptation by Deyo [24].

### Identification and definition of RA cohorts

In 2002, biologics were first introduced for RA treatment in Taiwan. Those available for RA treatment in Taiwan include TNF-α inhibitors (etanercept the first, followed by adalimumab) and rituximab before Dec. 2011. Golimumab, interleukin (IL)-6 receptor antibody (tocilizumab) and T-cell co-stimulatory modulator (abatacept) were not available until mid-2012. Small molecules inhibitors such as tofacitinib were only available after Oct. 2014. Two approved TNF-α inhibitors, infliximab and certolizumab, are not available in Taiwan. Therefore, golimumab, infliximab, certolizumab, tocilizumab, abatacept, or tofacitinib were not included in this study.

RA patients started TNF-α inhibitors or rituximab therapy according to the guidelines [25]. A total of 3,577 patients received etanercept at a dose of 25 mg twice weekly and 1,678 patients received adalimumab at a dose of 40 mg every other week, all with concomitant methotrexate (MTX) at a stable dose of 7.5–15 mg weekly. Patients who had treatment switches among TNF-α inhibitors were not included. A total of 763 patients who were refractory to TNF-α inhibitors received rituximab therapy in a 6-month fixed repeat protocol (1000mg twice with 14-day interval) annually. Patients taking csDMARDs who had never received biologics were eligible for csDMARDs-exposed group (n = 36,162).

### Identification of LTBI using QFT-G assay

Considering that interferon (IFN)-γ release assays (IGRA) provide a higher specificity than TST in detecting LTBI within a BCG-vaccinated population, IGRA is preferred as a test for LTBI in RA patients in Taiwan, where BCG vaccination is implemented universally. In case the IGRA results were indeterminate, TST would be performed using the Mantoux method, in which a positive result was defined as an induration diameter ≧5mm after the intradermal injection of 2 tuberculin units of PPD RT-23 (Staten Serum Institute, Denmark) [26].

Rheumatoid arthritis patients were evaluated at baseline using a standardized interview, chest radiographs, and QuantiFERON-TB Gold In-Tube (QFT-G) assay before biologic therapy. QFT-G assay was performed according to the manufacturer’s instructions (Cellestis Ltd., Victoria, Australia). The positive result of QFT-G assay was defined as an IFN-γ level≧0.35 IU/ml in TB-specific antigens-stimulated wells after subtracting the level in the nil well according to the manufacturer’s recommendation. Positive QFT-G results indicated the presence of LTBI and the need for isoniazid prophylaxis (INHP) [27]. Since 2010, the guideline in Taiwan has recommended that LTBI screening would be carried out, and 9-month INHP be initiated one month prior to biologic therapy in the presence of LTBI.

### Main outcome measurements

Patients with active TB were defined as those having a new diagnosis of TB, ICD-9-CM codes (ICD-9-CM codes 011–018), and receiving anti-TB therapy [7, 21]. Therefore, the data regarding TB are reliable. The study endpoint was defined as newly-onset TB disease or death during the 11-year follow-up period (2001–2011). In this study, we excluded patients with a history of TB before RA index date. The age- and sex-matched non-RA controls (age ≥18 years) were selected from the NHIRD.

### Potential confounders

We used propensity score, which estimates the probability of developing new-onset TB, to reduce the potential confounders. The propensity score was estimated by the logistic regression based on demographic factors such as age at entry, sex and CCI.

### Statistical analysis

The data were presented as the mean±standard deviations (SD) for continuous variables, and proportions for categorical variables. The differences between continuous values were analyzed using the independent t test for continuous variables, and the chi-square test for categorical variables. The incidences of newly-diagnosed TB disease in RA cohorts and the non-RA group were calculated. The multivariate Cox proportional hazards model was adjusted for age, gender, the used dose of corticosteroids, and the presence of comorbidities, and then used to identify independent factors contributing to the development of TB disease in the RA-to-control cohort; the 95% confidence interval (CI) for each variable was determined. We calculated the incidence rate of TB for each biological agent therapy and estimated biologics-specific HRs using csDMARDs only (the largest group) as reference. Propensity score adjustment served as for further confirmation of such association. All analyses were conducted using SAS statistical software version 9.2 (SAS Institute, Inc., Cary, NC, USA). A *p-*value of <0.05 was considered statistically significant.

## Results

### Demographic characteristics of study cohorts

During the period from 2001 to 2011, a total of 42,180 eligible RA patients were identified and enrolled, with a mean follow-up time of 6.6 years, and a total of 168,720 non-RA subjects were matched for age and gender with the ratio of 1:4 (Fig 1). As illustrated in in Table 1, RA patients receiving biologic therapy were younger than those receiving csDMARDs. Compared with the non-RA subjects, the RA patients demonstrated a significantly higher proportion of corticosteroids use (≧5mg/day) and took higher daily dose of corticosteroids. The RA patients also had significantly higher prevalence of comorbidities than the non-RA subjects (57.0% vs. 33.3%), including liver cirrhosis (8.8% vs. 6.5%), COPD (9.8% vs. 7.5%), and CKD (2.1% vs. 1.9%). Among the RA patients, the proportion of corticosteroids use (≧5mg/day) and the dosage were even higher in those receiving biologics than in those receiving csDMARDs only.

**Table 1:** Baseline characteristics and the occurrence of tuberculosis (TB) in rheumatoid arthritis (RA) patients and age- and gender-matched individuals from the general population 2001–2011 (n = 210,900).

	Non-RA	Total RA	RA with	RA with	RA with	RA with
	group	group	csDMARDs	Etanercept	Adalimumab	Rituximab
	(n = 168,720)	(n = 42,180)	(n = 36,162)	(n = 3,577)	(n = 1,678)	(n = 763)
Age, years, mean±SD	53.0 ± 14.6	53.4 ± 13.9	54.1 ± 14.0**	49.5 ± 12.6^##^	49.9 ± 12.5 ^##^	49.7 ± 12.1^##^
Female, n (%)	132,568 (78.6%)	33,142 (78.6%)	28,230 (78.1%)	2,933(82.0%)	1,339(79.8%)	640 (83.9%)
CCI≧1, n (%)	56,180 (33.3%)	24,059(57%)**	20,630(57.1%)**	2,075 (58%)	905 (53.9%)	449 (58.9%)
Comorbidity						
Diabetes mellitus	14,617 (8.7%)	3,518 (8.3%)	3,153 (8.7%)	203 (5.7%)^##^	121 (7.2%)	41 (5.4%)^#^
Liver cirrhosis	10,878 (6.5%)	3,720 (8.8%)**	3,287 (9.1%)**	270 (7.6%)^#^	106 (6.3%)^#^	57 (7.5%)
COPD	12,720 (7.5%)	4,124 (9.8%)**	3,630 (10%)**	308 (8.6%)^#^	121 (7.2%)^#^	65 (8.5%)
CKD	3,131 (1.9%)	864 (2.1%)*	790 (2.2%)**	37 (1.0%)^##^	19 (1.1%)^#^	18 (2.4%)
Use of corticosteroids at daily dose≧5mg						
Yes	1,159 (0.7%)	8,372 (19.9%)**	6,203 (17.2%)	1,221 (34.1%)^##^	1,071 (63.9%)^##^	422 (55.3%)^##^
No	167,561 (99.3%)	33,808 (80.2%)	29,959 (82.9%)	2,356 (65.9%)	607 (36.2%)	341 (44.7%)
Dose of corticosteroids (mg/day, mean ± SD)	0.2 ± 0.9	2.6 ± 3.2**	2.4 ± 3.1**	3.9 ± 3.4^##^	4.2 ± 3.6^##^	4.8 ± 3.4^##^
New-onset TB, n (%)	2,553 (1.5%)	1,103 (2.6%)**	913 (2.5%)**	121 (3.4%)^#^	67 (4.0%)^##^	2 (0.3%)
Location of TB						
Pulmonary TB	2,388 (93.5%)	982 (89.0%)**	820 (89.8%)*	102 (84.3%)	58 (86.6%)	2 (100%)
E-P TB	165 (6.5%)	121 (11.0%)**	93 (10.2%)*	19 (15.7%)*	9 (13.4%)*	0 (0.0%)
Drug-TB interval, yrs	NA	5.8 ± 3.7	6.8 ± 3.7	3.6 ± 2.4^##^	1.8 ± 1.2^##^	1.6 ± 0.9^##^
Follow-up period, yrs	10.2 ± 2.3	6.6 ± 3.7**	6.4 ± 3.7**	8.1 ± 3.1^##^	7.7 ± 3.3^##^	6.7 ± 3.5^#^

csDMARDs: conventional synthetic disease-modifying antirheumatic drugs; CCI: Charlson comorbidity index; COPD: chronic obstructive pulmonary disease; CKD: chronic kidney disease; E-P: extra-pulmonary; NA: not applicable; yrs: years.

* p<0.01,

** p<0.001, versus Non-RA group;

^#^ p<0.01,

^##^ p<0.001, versus RA with csDMARDs group

Among subjects with active TB, 121 (11.0%) of 1,103 TB-infected RA patients had extrapulmonary TB, which was significantly higher than that in non-RA group (6.5%, p<0.001). A significantly higher proportion of extrapulmonary TB was also observed in RA patients receiving therapy with TNF-α inhibitors (15.7% for etanercept and 13.4% for adalimumab) compared to the non-RA group (6.5%).

### Incidence rates and risk factors of TB in RA cohorts

We demonstrated that 1,103 RA patients developed TB in 276,476 person-years (py) of follow-up (incidence rate [IR] = 399 per 100,000 py) while 2,553 non-RA subjects contracted TB in 1,720,860 py (IR = 148 per 100,000 py). The incidence of TB was 2.7 times greater in RA cohort than in the non-RA group, with an adjusted HR (aHR) of 2.58 (95% confidence interval [CI] 2.39–2.78, p<0.001).

As illustrated in Table 2, advanced age (age≧65years, aHR = 4.37), male gender (aHR = 1.87), and the use of corticosteroids (≧5mg/day, aHR = 2.70) were significant risk factors for developing TB in RA patients. In comorbidity analysis, RA patients with DM (aHR = 1.26), COPD (aHR = 1.51), and CKD (aHR = 1.44) had significantly higher risks of developing TB compared to the non-RA group.

**Table 2:** Risk factors for developing TB adjusted by age at entry, sex, the use of corticosteroids, comorbidities and Charlson comorbidity index (CCI) in rheumatoid arthritis cohort.

Risk factors	Adjusted HR	95% CI	p-value
Age at entry, years			
18–44	1.00 (reference)	-	-
45–64	2.16	1.81–2.14	<0.0001
≧65	4.37	1.91–2.44	<0.0001
Gender			
Female	1.00 (reference)	-	-
Male	1.87	1.70–2.05	<0.0001
Use of corticosteroids			
No	1.00 (reference)	-	-
Yes	1.88	1.69–2.10	<0.0001
Use of corticosteroids			
Daily dose <5mg	1.00 (reference)	-	-
Daily dose≧5mg	2.70	2.43–3.01	<0.0001
CCI			
0	1.00 (reference)	-	-
1	1.23	1.12–1.34	<0.0001
2	1.22	1.09–1.36	0.0007
≧3	1.21	1.06–1.39	0.006
Comarbidity			
Diabetes mellitus	1.26	1.14–1.40	<0.0001
Liver cirrhosis	0.93	0.83–1.05	0.248
COPD	1.51	1.38–1.66	<0.0001
CKD	1.44	1.08–1.91	0.013

HR: hazard ratio; 95%CI: 95% confidence interval; COPD: chronic obstructive pulmonary disease; CKD: chronic kidney disease.

### Hazard ratios of TB in RA patients receiving different therapeutic agents

Among RA patients, csDMARDs-exposed patients were the largest treatment group (Fig 1). A total of 913 TB cases were identified in 231,759 csDMARDs-exposed py (IR = 394 per 100,000py) with a higher risk of TB (aHR = 2.47, 95% CI 2.29–2.67, p<0.001) compared with non-RA group. Using csDMARDs-exposed patients as a reference group, the aHR of TB was highest for adalimumab (1.52, 95% CI 1.18–1.96, p<0.005), followed by etanercept (1.16, 95% CI 0.95–1.41) (Table 3). Only two TB cases was identified in 6,179 rituximab-exposed py (aHR = 0.08, 95% CI 0.02–0.31, p<0.001).

**Table 3:** Incidence rates (IRs) and hazard ratios (HRs) of tuberculosis (TB) disease by biologic exposure with csDMARDs-exposed patients as reference group, and by isoniazid prophylaxis therapy (INHP) with the absence of INHP as reference group.

	TB cases/py	Crude IR per100000 py	Crude HR(95%CI)	Adjusted HR (95% CI)
csDMARDs-exposed	913/231,759	394	1.00 (reference)	1.00 (reference)
INHP (-)(n = 36,148)#	913/231,697	394	1.00 (reference)	1.00 (reference)
INHP (+) (n = 14)	0/62	0	NA	NA
Adalimumab-exposed	67/11,171	600	1.52(1.19–1.95)**	1.52(1.18–1.96)*
INHP (-) (n = 1,615)#	66/10,713	616	1.00 (reference)	1.00 (reference)
INHP (+) (n = 63)	1/459	218	0.35(0.05–2.50)	0.45(0.06–3.24)
Etanercept-exposed	121/27,367	442	1.14(0.94–1.37)	1.16(0.95–1.41)
INHP (-) (n = 3,508)#	121/26,880	450	1.00 (reference)	1.00 (reference)
INHP (+) (n = 69)	0/487	0	NA	NA
Rituximab-exposed	2/6,179	32	0.08(0.02–0.34)**	0.08(0.02–0.31)**
INHP (-) (n = 755)#	2/6,119	33	1.00 (reference)	1.00 (reference)
INHP (+) (n = 8)	0/60	0	NA	NA

^**#**^ Not indicative of total number of patients without latent tuberculosis infection

csDMARDs: conventional synthetic disease-modifying anti-rheumatic drugs; py: person-years; IR: incidence rate; HR: hazard ratio; 95% CI: 95% confidence interval; NA: not applicable; INHP(+): with isoniazid prophylaxis; INHP(+): without isoniazid prophylaxis. Adjusted HR estimated from multivariate Cox proportional hazard models comparing each biologic to csDMARDspropensity score adjusted for age, sex, the use of corticosteroids (daily dose≧5mg), and Charlson comorbidity index.

* p<0.005,

** p<0.001, versus tsDMARDs-exposed patients

The mean interval between drug exposure and TB development was the shortest for adalimumab (1.8±1.2 years), followed by etanercept (3.6±2.4 years), and the longest for csDMARDs-exposured patients (6.8±3.7 years, both p<0.001) (Table 1).

### The effectiveness of isoniazid prophylaxis on TB risk

As illustrated in Table 3, a total of 154 RA patients received INHP treatment, and only one subject had TB in adalimumab-exposed patients. None of csDMARDs-exposed, etanercept-exposed, or rituximab-exposed patients who received INHP developed TB. In addition, adalimumab-exposed patients who received INHP had a lower risk of developing TB (aHR = 0.45) compared to those without INHP.

**Fig 2:**
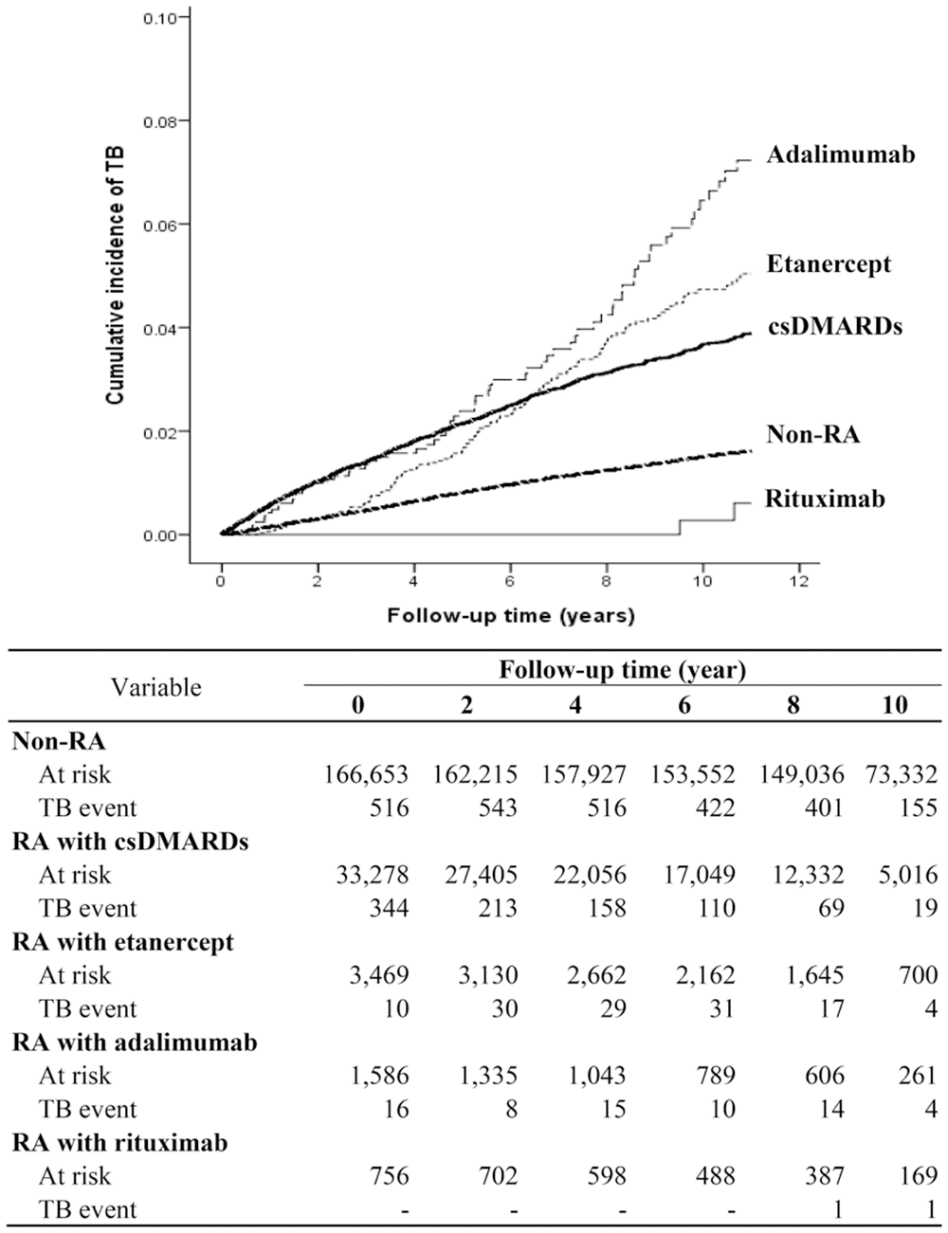
Cumulative incidence of tuberculosis in RA patients, who received therapy with csDMARDs, etanercept, adalimumab or rituximab, in comparison with non-RA subjects. csDMARDs: conventional synthetic disease-modifying anti-rheumatic drugs.

Kaplan-Meier analysis showed that the cumulative incidence of TB disease was the highest in adalimumab-exposed patients, followed by etanercept-exposed patients, csDMARDs-exposed patients, and the lowest in rituximab-exposed patients as well as non-RA subjects (Fig 2). The number of active TB rapidly declined after initiation of LTBI screening and INHP in Taiwan (at year 9 of follow-up period, 2010).

### Mortality after the diagnosis of TB in RA cohorts

As illustrated in Table 4, RA patients with TB infection had significantly higher death rates (aHR = 1.25) compared with the non-RA group. Advanced age (age≧65years, aHR = 4.31), the use of corticosteroids (≧5mg/day, aHR = 1.69), and the presence of DM (aHR = 1.16) were significant risk factors for mortality after TB diagnosis in RA patients. Using csDMARDs-exposed group as reference, death rate after TB diagnosis was significantly lower in adalimumab-exposed patients (aHR = 0.22, p<0.01), while no significant difference in death rate in etanercept-exposed patients. No mortality after TB diagnosis occurred in rituximab-exposed patients.

**Table 4:** Multivariable analysis for mortality after the diagnosis of tuberculosis (TB) in RA patients according to therapeutic agents and the risk factors of mortality.

Risk factors	Adjusted HR	95% CI	p-value
RA/Non-RA	1.25	1.11–1.40	<0.001
Therapeutic agents			
csDMARDs	1.00(reference)	-	-
Adalimumab	0.22	0.07–0.69	<0.01
Etanercept	1.08	0.76–1.54	0.669
Rituximab	NA	NA	NA
Age at entry, years			
18–44	1.00(reference)	-	-
45–64	2.36	1.70–3.28	<0.001
≧65	4.31	3.07–6.05	<0.001
Gender			
Female	1.00(reference)	-	-
Male	1.00	0.92–1.09	0.944
Use of corticosteroids			
Daily dose <5mg	1.00 (reference)	-	-
Daily dose≧5mg	1.69	1.47–1.95	<0.001
CCI			
0	1.00(reference)	-	-
1	0.97	0.85–1.12	0.690
2	1.05	0.89–1.25	0.571
≧3	1.08	0.89–1.32	0.428
Comarbidity			
Diabetes mellitus	1.16	1.00–1.34	<0.05
Liver cirrhosis	1.05	0.88–1.25	0.617
COPD	0.91	0.80–1.04	0.176
CKD	1.10	0.77–1.59	0.593

csDMARDs: conventional synthetic disease-modifying anti-rheumatic drugs; HR: hazard ratio; 95%CI: 95% confidence interval; CCI: Charlson comorbidity index; COPD: chronic obstructive pulmonary disease; CKD: chronic kidney disease.

Adjusted HR estimated from multivariate Cox proportional hazard models comparing each biologic to csDMARDspropensity score adjusted for age, sex, and Charlson comorbidity index.

## Discussion

The prevention of TB relies heavily on early identification, prevention of LTBI reactivation, and infection control measures [1, 27]. While RA patients were shown to have elevated TB risk [5–7], the causes are still not completely known. We performed this nationwide population-based cohort study to investigate the impact of different therapeutic agents on TB development and mortality rates after TB diagnosis in RA patients, as well as their risk factors in real-world settings. We also evaluated the effectiveness of INHP therapy in biologics–associated TB prevention. Resonating with previous studies [5–7], we demonstrated that RA patients had a 2.7-fold increased risk of developing TB compared with the non-RA group. Advanced age (≥ 65 years), male gender, the use of corticosteroids, and the presence of comorbidities including DM, COPD and CKD were all significant risk factors for developing TB in the RA cohort. Using the csDMARDs-exposed group as a reference, TNF-α inhibitors posed a significantly higher TB risk, in contrast to rituximab which was associated with a significantly lower TB risk. INHP could effectively reduce TB risk in adalimumab-exposed patients (aHR = 0.45) compared with those without INHP.

Similar to Taiwan Centers for Disease Control (CDC) reports [4], our results showed that advanced age and male gender were major risk factors for developing TB. We also demonstrated that the use of corticosteroids was a significant risk factor for developing TB in RA patients, in agreement with the results reported by Jick *et al*. [11]. Our RA patients with comorbidities including DM, COPD, and CKD had significantly higher risk of developing TB compared with those without comorbidities, which was consistent with the findings that DM, COPD, and CKD are risk factors for active TB [9, 28–29]. Therefore, multidisciplinary team care should be a mandatory strategy for preventing TB development in RA patients with comorbidities.

Given that T-cell immunity and the phagocytic activities of macrophages are crucial defense mechanisms against mycobacterial infection [30], the immunosuppressive effect of csDMARDs contributes to the elevated TB risk in RA patients [14, 31–33]. Our results showed a 2.5-fold (aHR = 2.47) increase of TB risk in csDMARDs-exposed patients compared with the non-RA subjects, similar to previous findings [14, 32–33]. Using the csDMARDs-exposed group as a reference, we found that the adalimumab-exposed patients had even higher TB risk, suggesting a TNF-blocking effect on TB risk [14, 34–36]. In addition, the aHR and cumulative incidence of TB disease were higher in the adalimumab-exposed versus the etanercept-exposed patients, consistent with previous findings of a higher TB risk associated with monoclonal antibody treatment versus soluble receptor anti-TNF-α therapy [14, 37–38]. Furthermore, the interval between drug exposure and TB onset was the shortest in the adalimumab-exposed (mean, 1.8 years), followed by the etanercept-exposed (3.6 years), and the longest in the csDMARDs-exposed patients. Although the risk of developing active TB due to the reactivation of LTBI was highest during the first 6–12 months of anti-TNF-α therapy, there was also a possibility of *de novo* re-infection with *Mycobacterium tuberculosis* in an area with high or intermediate TB burden like Taiwan. Therefore, we can’t differentiate between LTBI reactivation and new TB infection in spite that the time interval of drug exposure to TB development varied among different biologics.

In the present study, only two TB cases were identified in 6,179 rituximab-exposed py with a significantly lower IR compared with the non-RA group (32 vs. 148 per 100,000 py), and a lower TB risk (aHR = 0.08) compared with the csDMARDs group. Rituximab is a monoclonal antibody directed against B-cell marker CD20, playing a much less role in the immunity against *M*. *tuberculosis* infection. Our previous data showed no significant effect of rituximab therapy on the released IFN-γ levels in RA patients with LTBI [19]. Such results resonated with previous studies which showed no increase in TB risk in RA patients treated with rituximab in clinical trials and daily practice [14,18–19], and supported the long-term safety of rituximab treatment [39]. However, the majority of our rituximab-exposed patients had history of previous anti-TNF-α therapy, which may result in a population bias (patients might have dropped out due to previous reactivation of LTBI following anti-TNF-α therapy).

Guidelines have recommended that effective LTBI screening should be routinely carried out and prophylactic therapy be initiated before starting biologic therapy if LTBI exists [15]. The majority of biologics–associated TB cases were related to improper implementation of LTBI screening and prophylaxis [40]. Since the incidence rate of TB is high and the protective effect of BCG vaccination against TB remains controversial in Taiwan, identification and treatment of LTBI in high risk groups becomes an important part of TB prevention strategy. In the present study, the number of active TB cases have declined after implementing the policy of LTBI screening and INHP in Taiwan since 2010 (Fig 2). In addition, we demonstrated that INHP could effectively reduce TB risk in RA patients treated with biologics, which was also shown in other previous studies [15, 40–41] and our hospital-based study [3]. These observations support the findings of recent studies that preventive chemotherapy could effectively reduce TB risk among IGRAs-positive contacts or rheumatic patients before starting anti-TNF-α therapy [40, 42]. Although our data showed the effectiveness of INHP for biologics-exposed patients, the total incidence of TB was still increased in patients with anti-TNF-α therapy. We thought the discrepancy was caused by incomprehensive screening of LTBI and the ensuing under-diagnoses of LTBI cases, which was also reflected in the findings that only 2.5% of 6018 biologics-treated patients received INHP in the present study.

Although the local guideline has recommended that LTBI screening should be carried out before biologics treatment for RA patients, it is still not a mandatory routine examination, and LTBI cases need not be reported to the public health administration in Taiwan. In addition, because of the low specificity of TST and high cost of IGRAs, there were very few hospitals which can fully conducted LTBI screening before biologics treatment for RA patients in Taiwan. Based on those reasons, the real epidemiological data of LTBI are not available. According to the registry data in one medical center, 240 (23.6%) of 1,025 biologic exposed patients with RA had positive QFT-G results before starting biologic therapy. One of our previous studies also revealed that 45 (18.6%) of 242 RA patients had positive QFT-G results in the LTBI screening [3], which was similar to another study in Korea (17.0%) [43]. Fully conducted LTBI screening and stricter implementation of INHP, suggested by a medical center-based study, would be beneficial for biologics-related TB prevention [3]. Further confirmation through larger studies is required in the future.

Among the epidemic infectious diseases in Taiwan, TB is associated with the highest mortality rate (2.8 cases per 100,000 people in 2012) [4], which were higher than in other Asian (e.g. Japan and Korea) or Western countries. Similar to some recent studies [44–46], a significantly higher death rate after developing TB was observed in our RA patients, particularly the elderly and those with DM, compared with non-RA control. It is interesting that the death rate was significantly lower in our adalimumab-exposed patients in comparison to the csDMARDs-exposed patients. We considered it was the results of increased awareness of and alertness to TB disease among clinicians, with more thorough assessment for possible signs of active TB for biologics-exposed patients.

There are several limitations in this study. The Taiwan NHIRD does not contain detailed information about lifestyle factors or individual health status (e.g. body mass index, malnutrition) that may be related to TB infection [11]. In addition, NHIRD does not include the results of laboratory examinations such as IGRA. Because LTBI screening was not a mandatory examination for RA patients scheduled for biologic therapy before 2010, and LTBI cases do not have to be reported to the public health administration in Taiwan, the real epidemiological data for LTBI are not available in RA cohort. In order to improve the accuracy of TB diagnosis, we used not only the ICD-9-CM codes, but also anti-mycobacterial therapy receipts to identify TB cases. Because Taiwan lists TB as a type 3 notifiable disease, of which every newly-diagnosed case is required to be reported within a week to the Taiwan Centers for Disease Control, the data regarding TB diagnosis were reliable.

The major strength of this study was the utilization of nationwide database which provides detailed medical care records and is widely accepted as an instrument for epidemiological studies [7, 21]. The NHIRD contains detailed pharmacy claims for each study subject, with the diagnoses based on the ICD-9-CM, registration files, and original claims records for reimbursement. The large sample size of the NHIRD (23 million enrollees) and the long-term records enhanced the statistical power and accuracy of this study. Moreover, we directly compared the risk of TB and mortality between csDMARDs-exposed and biologics-exposed patients.

## Conclusion

TB risk was increased in RA patients, particularly in those receiving TNF-α inhibitors, but nevertheless the risk associated with rituximab therapy was relatively low, with the aHR of which was even lower than that associated with csDMARDs. In addition, advanced age, the use of corticosteroids (≧5mg/day), and the presence of comorbidities were the significant risk factors for both developing TB and mortality after TB diagnosis in RA patients. A high index of suspicion and prompt diagnosis of active TB are necessary for the prevention of TB-related mortality in RA patients.

## References

[1] World Health Organization. Global tuberculosis report 2014. Available: http://www.who.int/tb/publications/global_report/en/. 2014.

[2] Jou R, Huang WL, Su WJ. Tokyo-172 BCG vaccination complications, Taiwan. Emerg Infect Dis 2009;15:1525–1526. 10.3201/eid1509.081336 19788832 PMC2819856

[3] Chen DY, Shen GH, Chen YM, Chen HH, Hsieh CW, Lan JL. Biphasic emergence of active tuberculosis in rheumatoid arthritis patients receiving TNFα inhibitors: The utility of IFNγ assay. Ann Rheum Dis 2012;71:231–237. 10.1136/annrheumdis-2011-200489 22021896

[4] Centers for Disease Control, R.O.C. (Taiwan). Taiwan Tuberculosis Control Report 2013. 2013.

[5] Doran MF, Crowson CS, Pond GR, O'Fallon WM, Gabriel SE. Frequency of infection in patients with rheumatoid arthritis compared with controls: a population-based study. Arthritis Rheum 2002;46:2287–93. 12355475 10.1002/art.10524

[6] Yamada T, Nakajima A, Inoue E, Tanaka E, Hara M, Tomatsu T, et al Increased risk of tuberculosis in patients with rheumatoid arthritis in Japan. Ann Rheum Dis 2006;65:1661–1663. 16837491 10.1136/ard.2005.047274 PMC1798455

[7] Liao TL, Lin CH, Shen GH, Chang CL, Lin CF, Chen DY. Risk for mycobacterial disease among patients with rheumatoid arthritis, Taiwan, 2001–2011. Emerg Infect Dis. 2015;21:1387–1395. 10.3201/eid2108.141846 26196158 PMC4517709

[8] Dougados M, Soubrier M, Antunez A, Balint P, Balsa A, Buch MH, et al Prevalence of comorbidities in rheumatoid arthritis and evaluation of their monitoring: Results of an international, cross-sectional study (COMORA). Ann Rheum Dis 2014;73:62–68. 10.1136/annrheumdis-2013-204223 24095940 PMC3888623

[9] Lobue P, Menzies D. Treatment of latent tuberculosis infection: an update. Respirology 2010;15:603–622. 10.1111/j.1440-1843.2010.01751.x 20409026

[10] Marais BJ, Lonnroth K, Lawn SD, Migliori GB, Mwaba P, Glaziou P, et al Tuberculosis comorbidity with communicable and non-communicable diseases: Integrating health services and control efforts. Lancet Infect Dis 2013;13:436–448. 10.1016/S1473-3099(13)70015-X 23531392

[11] Jick SS, Lieberman ES, Rahman MU, Choi HK. Glucocorticoid use, other associated factors, and the risk of tuberculosis. Arthritis Rheum 2006;55:19–26. 16463407 10.1002/art.21705

[12] Gomez-Reino JJ, Carmona L, Valverde VR, Mola EM, Montero MD. Treatment of rheumatoid arthritis with tumor necrosis factor inhibitors may predispose to significant increase in tuberculosis risk: A multicenter active-surveillance report. Arthritis Rheum 2003;48:2122–2127. 12905464 10.1002/art.11137

[13] Gardam MA, Keystone EC, Menzies R, Manners S, Skamene E, Long R, et al Anti-tumour necrosis factor agents and tuberculosis risk: Mechanisms of action and clinical management. Lancet Infect Dis 2003;3:148–155. 12614731 10.1016/s1473-3099(03)00545-0

[14] Arkema EV, Jonsson J, Baecklund E, Bruchfeld J, Feltelius N, Askling J. Are patients with rheumatoid arthritis still at an increased risk of tuberculosis and what is the role of biological treatments? Ann Rheum Dis 2015;74:1212–7. 10.1136/annrheumdis-2013-204960 24608401

[15] Carmona L, Gomez-Reino JJ, Rodriguez-Valverde V, Montero D, Pascual-Gomez E, Mola EM, et al Effectiveness of recommendations to prevent reactivation of latent tuberculosis infection in patients treated with tumor necrosis factor antagonists. Arthritis Rheum 2005;52:1766–1772. 15934089 10.1002/art.21043

[16] Cohen SB, Emery P, Greenwald MW, Dougados M, Furie RA, Genovese MC, et al for the REFLEX trial group. Rituximab for rheumatoid arthritis refractory to anti-tumour necrosis factor therapy. Arthritis Rheum 2006; 54:2793–806. 16947627 10.1002/art.22025

[17] Maglione PJ, Xu J, Chan J. B cells moderate inflammatory progression and enhance bacterial containment upon pulmonary challenge with Mycobacterium tuberculosis. J Immunol 2007; 178:7222–34. 17513771 10.4049/jimmunol.178.11.7222

[18] Edwards JC, Szczepanski L, Szechinski J, Filipowicz-Sosnowska A, Emery P, Close DR, et al Efficacy of B-cell-targeted therapy with rituximab in patients with rheumatoid arthritis. N Engl J Med 2004; 350:2572–81. 15201414 10.1056/NEJMoa032534

[19] Chen YM, Chen HH, Lai KL, Hung WT, Lan JL, Chen DY. The effects of rituximab therapy on released interferon-gamma levels in the QuantiFERON assay among RA patients with different status of Mycobacterium tuberculosis infection. Rheumatology (Oxford) 2013; 52:697–704. 23264552 10.1093/rheumatology/kes365

[20] Winthrop KL, Yamashita S, Beekmann E, Polgreen PM, on behalf of the Infectious diseases Society of America Emerging Infections Network. Mycobacterial and other serious infections in patients receiving anti-tumor necrosis factor and other newly approved biologic therapies: case finding through the emerging infections network. Clin Infect Dis 2008; 46:1738–1740. 10.1086/587989 18419421

[21] Lee CH, Lee MC, Lin HH, Shu CC, Wang JY, Lee LN, et al Pulmonary tuberculosis and delay in anti-tuberculous treatment are important risk factors for chronic obstructive pulmonary disease. PLoS One 2012;7:e37978 10.1371/journal.pone.0037978 22662259 PMC3360660

[22] The National Health Insurance Statistics. Available: http://www.nhi.gov.tw/English/webdata/webdata.aspx?menu=11&menu_id=296&webdata_id=1942&WD_ID=296].

[23] Arnett FC, Edworthy SM, Bloch DA, McShane DJ, Fries JF, Cooper NS, et al The American Rheumatism Association 1987 revised criteria for the classification of rheumatoid arthritis. Arthritis Rheum 1988;31:315–324. 3358796 10.1002/art.1780310302

[24] Deyo RA, Cherkin DC, Ciol MA. Adapting a clinical comorbidity index for use with icd-9-cm administrative databases. J Clin Epidemiol 1992;45:613–619. 1607900 10.1016/0895-4356(92)90133-8

[25] Ledingham J, Deighton C. Update on the British Society for Rheumatology guidelines for prescribing TNFα blockers in adults with rheumatoid arthritis (update of previous guidelines of April 2001). Rheumatology (Oxford) 2005;44:157–163. 15637039 10.1093/rheumatology/keh464

[26] American Thoracic Society. Diagnostic standards and classification of tuberculosis in adults and children. *Am J Respir Crit Care Med* 2000;161:1376–1395. 10764337 10.1164/ajrccm.161.4.16141

[27] Lee H, Park HY, Jeon K, Jeong BH, Hwang JW, Lee J, et al QuantiFERON-TB Gold In-Tube assay for screening arthritis patients for latent tuberculosis infection before starting anti-tumor necrosis factor treatment. PLoS One 2015;10:e0119260 10.1371/journal.pone.0119260 25746854 PMC4352032

[28] Schluger NW. Challenges of treating latent tuberculosis infection. Chest 2002;121:1733–1735. 12065329 10.1378/chest.121.6.1733

[29] Doran MF, Crowson CS, Pond GR, O'Fallon WM, Gabriel SE. Predictors of infection in rheumatoid arthritis. Arthritis Rheum 2002;46:2294–2300. 12355476 10.1002/art.10529

[30] Gupta A, Kaul A, Tsolaki AG, Kishore U, Bhakta S. Mycobacterium tuberculosis: immune evasion, latency, and reactivation. Immunobiology 2012;217:363–74. 10.1016/j.imbio.2011.07.008 21813205

[31] Brode SK, Jamieson FB, Ng R, Campitelli MA, Kwong JC, Paterson JM, et al Risk of mycobacterial infections associated with rheumatoid arthritis in Ontario, Canada. Chest 2014;146:563–572. 10.1378/chest.13-2058 24384637

[32] Brassard P, Lowe AM, Bernatsky S, Kezouh A, Suissa S. Rheumatoid arthritis, its treatments, and the risk of tuberculosis in Quebec, Canada. Arthritis Rheum 2009;61:300–4. 10.1002/art.24476 19248128

[33] Kourbeti IS, Ziakas PD, Mylonakis E. Biologic therapies in rheumatoid arthritis and the risk of opportunistic infections: A meta-analysis. Clin Infect Dis 2014;58:1649–1657. 10.1093/cid/ciu185 24647016

[34] Askling J, Fored CM, Brandt L, Baecklund E, Bertilsson L, Coster L, et al Risk and case characteristics of tuberculosis in rheumatoid arthritis associated with tumor necrosis factor antagonists in Sweden. Arthritis Rheum 2005;52:1986–1992. 15986370 10.1002/art.21137

[35] Winthrop KL, Iseman M. Bedfellows: Mycobacteria and rheumatoid arthritis in the era of biologic therapy. Nat Rev Rheumatol 2013;9:524–531. 10.1038/nrrheum.2013.82 23797309

[36] Winthrop KL, Baxter R, Liu L, Varley CD, Curtis JR, Baddley JW, et al Mycobacterial diseases and anti-tumour necrosis factor therapy in USA. Ann Rheum Dis 2013;72:37–42. 10.1136/annrheumdis-2011-200690 22523429

[37] Tubach F, Salmon D, Ravaud P, Allanore Y, Goupille P, Breban M, et al Risk of tuberculosis is higher with anti-tumor necrosis factor monoclonal antibody therapy than with soluble tumor necrosis factor receptor therapy: The three-year prospective French research axed on tolerance of biotherapies registry. Arthritis Rheum 2009;60:1884–1894. 10.1002/art.24632 19565495 PMC2921546

[38] Navarra SV, Tang B, Lu L, Lin HY, Mok CC, Asavatanabodee P, et al Risk of tuberculosis with anti-tumor necrosis factor-α therapy: Substantially higher number of patients at risk in Asia. Int J Rheum Dis 2014;17:291–298. 10.1111/1756-185X.12188 24131578 PMC4034594

[39] van Vollenhoven RF, Emery P, Bingham CO III, Keystone EC, Fleischmann RM, Furst DE, et al Long-term safety of rituximab in rheumatoid arthritis: 9.5-year follow-up of the global clinical trial programme with a focus on adverse events of interest in RA patients. Ann Rheum Dis 2013; 72:1496–1502. 10.1136/annrheumdis-2012-201956 23136242 PMC3756452

[40] Gomez-Reino JJ, Carmona L, Angel Descalzo M. Risk of tuberculosis in patients treated with tumor necrosis factor antagonists due to incomplete prevention of reactivation of latent infection. Arthritis Rheum 2007;57:756–761. 17530674 10.1002/art.22768

[41] World Health Organization. Global tuberculosis control: surveillance, planning, finances. Geneva, Switzerland: World Health Organization, 2009.

[42] Zellweger JP, Sotgiu G, Block M, Dore S, Altet N, Blunschi R, et al Risk assessment of tuberculosis in contacts by interferon-γ release assays (IGRAs). A TBNET study. Am J Respir Crit Care Med 2015 [Epub ahead of print] 10.1164/rccm.201502-0232OC 25763458

[43] Lee JH, Sohn HS, Chun JH, Kim HA, Suh CH, Lee YW, et al Poor agreement between QuantiFERON-TB Gold test and tuberculin skin test results for the diagnosis of latent tuberculosis infection in rheumatoid arthritis patients and healthy controls. *Korean J Intern Med* 2014;29(1):76–84. 10.3904/kjim.2014.29.1.76 24574836 PMC3932398

[44] Lin CH, Lin CJ, Kuo YW, Wang JY, Hsu CL, Chen JM, et al Tuberculosis mortality: patient characteristics and causes. BMC Infect Dis 2014; 14:5 10.1186/1471-2334-14-5 24387757 PMC3890594

[45] Yeh JJ, Wang YC, Sung FC, Kao CH. Rheumatoid arthritis increases the risk of nontuberculosis mycobacterial disease and active pulmonary tuberculosis. PloS One 2014; 9:e110922 10.1371/journal.pone.0110922 25337995 PMC4206451

[46] Reed GW, Choi H, Lee SY, Lee M, Kim Y, Park H, et al Impact of diabetes and smoking on mortality in tuberculosis. PLoS One 2013; 8:e58044 10.1371/journal.pone.0058044 23469139 PMC3585219

